# Dissolving Potassium Tablets Masquerading as Concealed Foreign Body Ingestion

**DOI:** 10.7759/cureus.20581

**Published:** 2021-12-21

**Authors:** Michael A Craig, Carl Kay, Thomas B Wells, Maurice C Barnes

**Affiliations:** 1 Internal Medicine, Brooke Army Medical Center, San Antonio, USA; 2 Gastroenterology, Brooke Army Medical Center, San Antonio, USA; 3 Gastroenterology, The University of Tennessee Health Science Center, Memphis, USA

**Keywords:** radio-opaque, deliberate foreign body ingestion, potassium chloride, non-intentional self-ingestion, image findings

## Abstract

Foreign body ingestion is a common consultation for gastroenterologists. Without knowing the object(s) ingested, the differential diagnosis is broad, especially in patients with underlying mental health conditions, such as uncontrolled bipolar disorder, prior suicide attempts, or recreational drug use. The differential should include substances taken with suicidal intent or for concealment of illicit drugs. Certain foreign objects may require urgent or emergent endoscopic intervention. However, one should also consider benign, iatrogenic causes such as large, radiolucent potassium pills given in the emergency department, which do not require further intervention or hospitalization.

## Introduction

Foreign body ingestion occurs frequently, and often results in patients seeking care at emergency facilities. Approximately 80% of ingestions can be managed conservatively, but 10-20% do require endoscopic intervention [1]. Of note, the subset of foreign objects that are intentionally ingested may have increased need for endoscopic intervention (63-76%) or surgical intervention (12-16%) [2,3]. These intentional ingestions can include sharp objects, pieces of glass, razor blades, or packets of illegal drugs. Many complications can arise from accidental or incidental ingestion of foreign bodies, such as complete obstruction of the esophagus, perforation of gastrointestinal viscera, or overdoses of consumed drugs [4]. For this reason, it is important that patients with ingestion are evaluated appropriately with a thorough history and physical examination, imaging, and subspecialty consultation if appropriate. The endoscopic management of ingested objects varies depending on the type of the object, the location of the object, and the timing of ingestion [5]. However, it is important to note that benign medications such as radiopaque tablets given in the emergency department (ED) may not require endoscopy [6,7]. Here we present a case of large potassium tablets mimicking ingestion of potentially lethal objects.

## Case presentation

**Figure 1:**
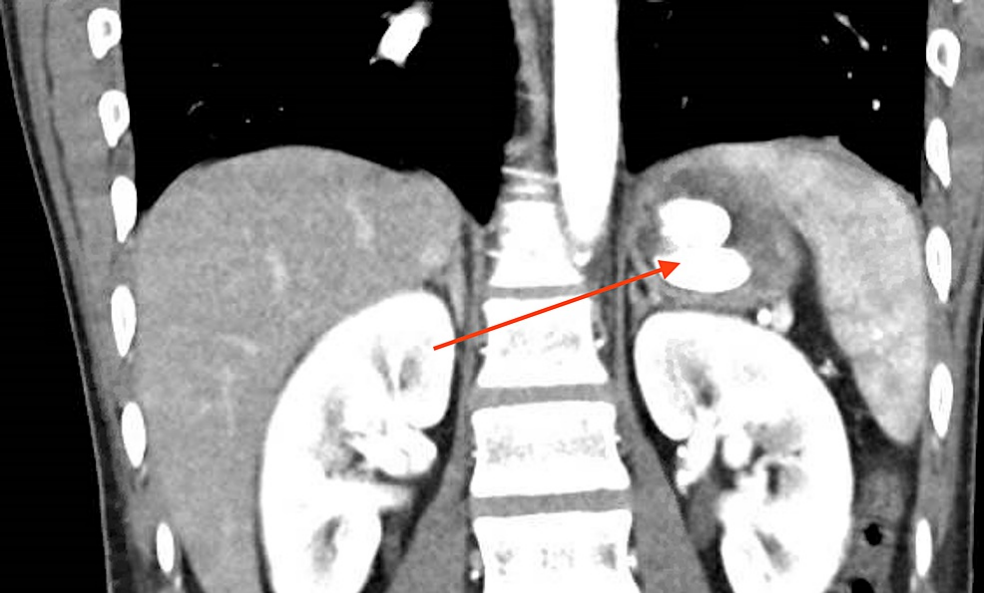
Two foreign bodies seen in the stomach on CT scan with red arrow indicating location CT, computed tomography

**Figure 2:**
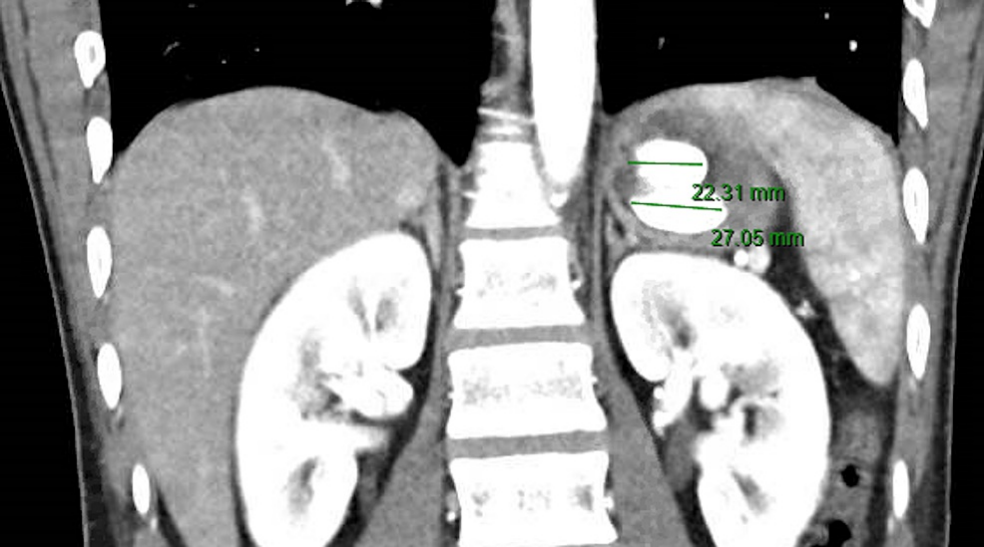
Two foreign bodies seen in the stomach on CT, measured via radiology software CT, computed tomography

A 25-year-old male with untreated bipolar disorder, substance abuse, and a prior suicide attempt was brought to the ED after a motor vehicle collision. He was an unrestrained passenger who was hit by another vehicle. In the trauma ED, he was evaluated for injuries with a computed tomography (CT) scan of his chest, abdomen, and pelvis. During the evaluation by the ED physician, he endorsed suicidal ideation. He was hemodynamically stable with unremarkable labs except for potassium of 2.9 mmol/L. CT scan showed no evidence of traumatic findings, obstruction, free air, or ascites. However, within the stomach there were two rounded hyperdense objects measuring approximately 2.5 x 1 x 1 cm, which could represent an ingested foreign body (Figures 1, 2).

Based on these findings, the patient was asked if he had ingested any foreign objects, which he denied. He was admitted for observation due to potential foreign body ingestion and suicidal ideation. Even though the patient denied any intentional ingestion, the leading diagnosis at presentation was ingestion of a package of drugs to conceal it from authorities. Gastroenterology was consulted at this time.

**Figure 3:**
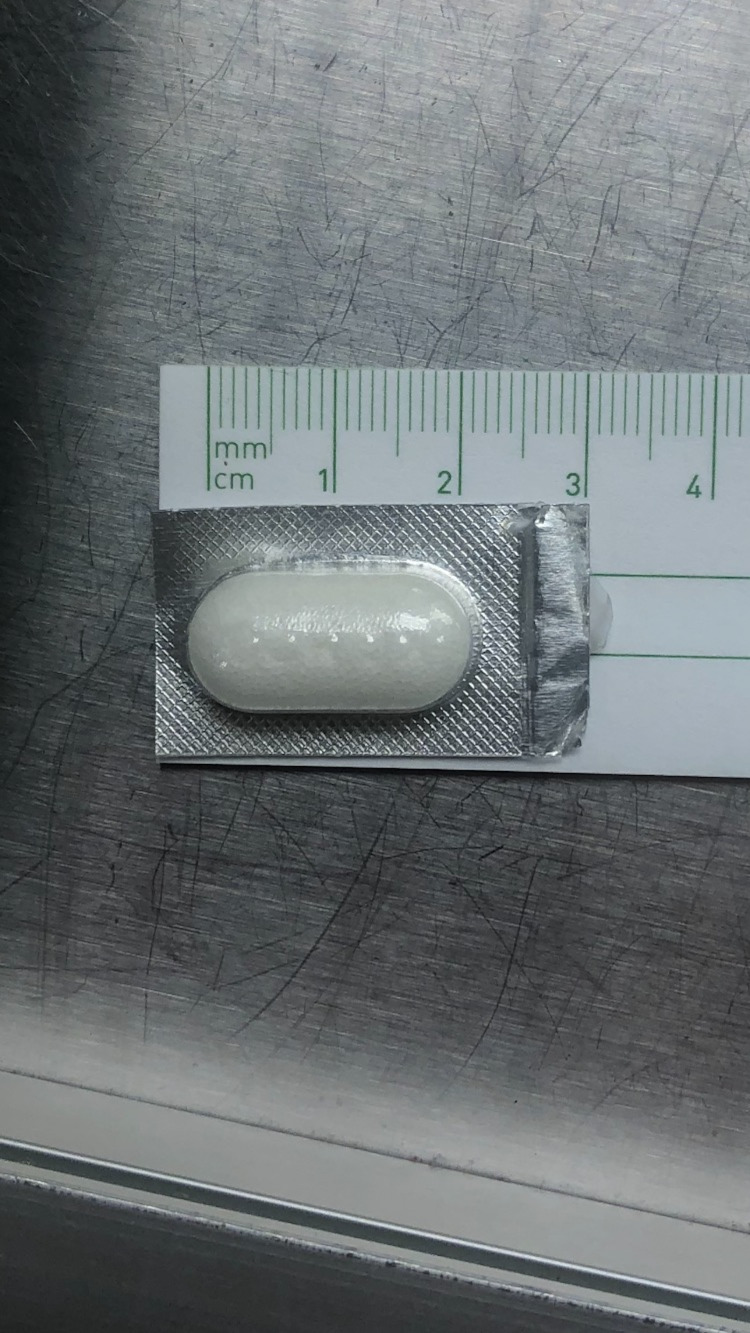
The potassium pills carried by the hospital, measuring 20 mm

**Figure 4:**
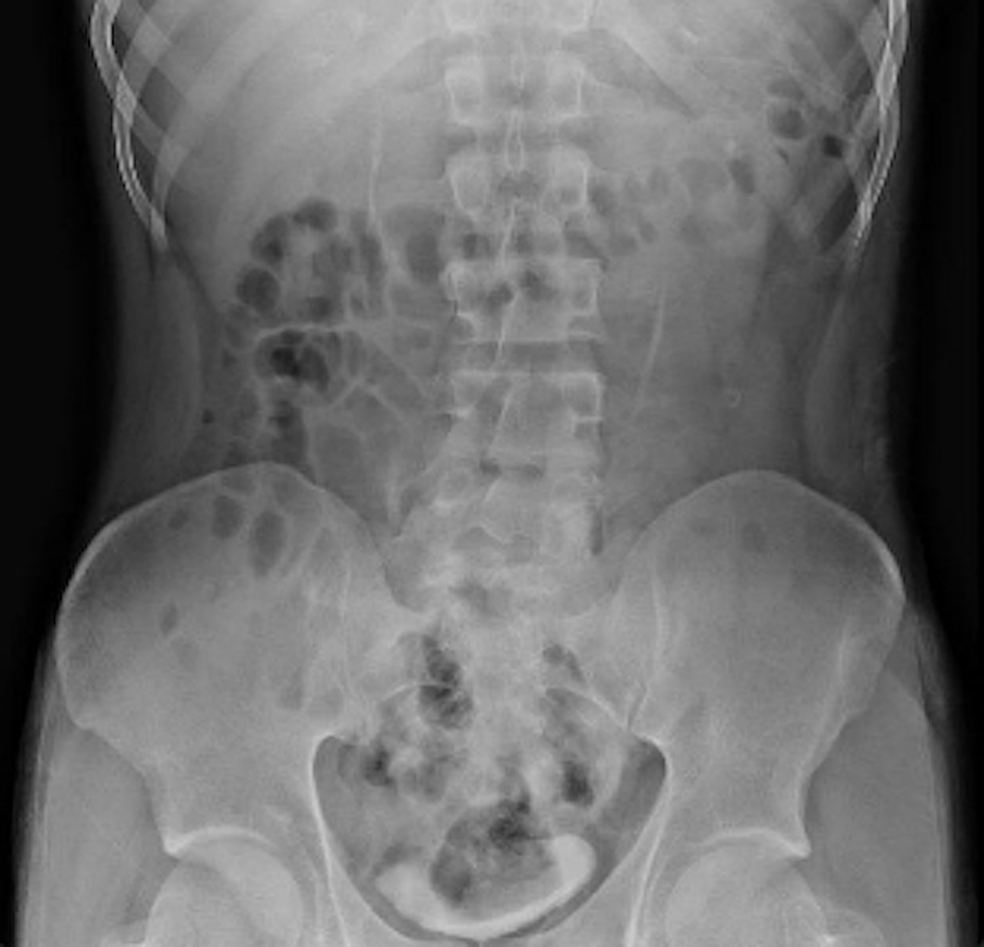
Unremarkable abdominal X-ray several hours after foreign bodies were seen on CT CT, computed tomography

On evaluation by the gastroenterology service, the patient continued to deny any foreign body ingestion. Review of the medication administration log showed he was given two 40 mEq KCl tablets 10 minutes prior to the CT scan for hypokalemia. After confirming with pharmacy, the KCl tablets at the institution measured 1 cm x 1 cm x 2 cm (Figure 3). Repeat abdominal X-rays a few hours after the CT scan showed no signs of a foreign body (Figure 4). The gastroenterology team determined that the foreign objects seen on CT scan were likely the two KCl tablets he had ingested just minutes before imaging. The patient was evaluated by a mental health provider and deemed safe for discharge by the gastroenterology service and a mental health provider without need for endoscopic intervention.

## Discussion

Foreign body ingestion is a reasonable indication for gastroenterology consultation, due to the potential need for endoscopic intervention emergently or urgently. Given the psychiatric comorbidities and possible motive for concealing illicit substances from authorities after the motor vehicle collision, foreign body ingestion was reasonable to include within the initial differential diagnosis in the context of the CT findings [4]. However, given the characteristics of the foreign body, there were no indications for emergent or urgent intervention, as the visualized object was blunt, medium sized, and located within the stomach (Tables 1, 2) [1]. It should also be noted that the European Society of Gastrointestinal Endoscopy Guidelines on Foreign Body Ingestion for 2016 recommend against endoscopic intervention of “drug packing” cases due to the risk of rupture and potential overdose, and recommend surgical referral in cases of packet rupture or obstruction [5].

**Table 1:** Management of foreign bodies Adapted from the 2016 ESGE guidelines [5]

Object type	Location	Timing
Sharp-pointed foreign body	Esophagus	Emergent
	Stomach/small bowel	Urgent
Blunt and small foreign body (<2 cm diameter)	Esophagus	Urgent
	Stomach/small bowel	Non-urgent
Blunt and medium foreign body (2-5 cm diameter)	Esophagus	Urgent
	Stomach/small bowel	Non-urgent
Blunt and large foreign body (>5 cm diameter)	Esophagus	Urgent
	Stomach/small bowel	Urgent

**Table 2:** Categories of ingested objects Adapted from the 2016 ESGE guidelines [5]

Type	Examples
Blunt	Coin, button, toy, packet of illegal drugs
Sharp-pointed	Needle, toothpick, bone, safety pin, glass, razor blades

Various other radiopaque pills can also masquerade as foreign bodies, including heavy metals, iron, and sustained-release medications [6]. Other medications that have been visualized as foreign bodies include theophylline, erythromycin, and multivitamins; of the medications tested in a 1998 Mayo Clinic study, potassium chloride is the most radio-dense (Table 3) [7]. Of note, an ingested potassium pill has also mimicked a hyperdense lesion suggestive of gastric bleeding on CT [8]. Other foreign bodies that can be incidentally found within the gastrointestinal lumen on imaging include bezoars, gallstones, endoscopic capsules, dentures, and batteries [9].

**Table 3:** Relative radiodensity of various medications Calculated by subtracting the background density from the density of the pill at its center. Adapted from Mayo Clinical Proceedings, 1998 [7]

Medication	Relative radiodensity
Potassium chloride	0.52
Ferrous sulfate	0.43
Calcium carbonate	0.35
Theophylline	0.20
Erythromycin	0.20
Amoxicillin-clavulanate	0.20
Clarithromycin	0.18

Our patient received two 20 mEq KCl tablets in the ED and was then found to have intragastric foreign bodies on CT. Repeat imaging was performed several hours later, which demonstrated complete resolution of the radiopaque objects. No overt passage of foreign bodies occurred in the stool while the patient was admitted. Because endoscopy was not pursued, the KCl tablets were never directly visualized within the gastric lumen. However, it is reasonable to assume that these tablets were the direct cause of the CT findings.

In summary, the temporal relationship between oral potassium repletion and imaging is an important consideration when confronted with unexpected foreign bodies found on non-invasive imaging studies, especially in patients with a past medical history of psychiatric illness or patients presenting to the hospital after contact with law enforcement.

## Conclusions

Foreign body ingestion is a common cause of emergency room visits. Ingestion of accidental and intentional foreign bodies such as obstructive food boluses, sharp objects, lethal doses of medication, or “drug packing” can cause serious complications requiring the need for emergent or urgent endoscopic or surgical interventions. These complications can include complete esophageal obstruction, perforation of abdominal organs, or overdose, but not every case requires gastrointestinal consultation or procedural intervention. It is possible for benign, radiopaque objects such as potassium chloride tablets to mimic these potentially lethal ingestions. We present this case to make other physicians aware that in the right setting, medication tablets should be considered in the differential for foreign body ingestion. As always, a thorough history from the patient and review of the electronic medical records can help identify these benign cases.
